# Recurrent urinary tract infection with antibiotic‐resistant *Klebsiella pneumoniae* in a patient with Crohn's disease: A case report

**DOI:** 10.1002/ccr3.4531

**Published:** 2021-08-11

**Authors:** Masoumeh Azimirad, Mercedeh Tajbakhsh, Abbas Yadegar, Mohammad Reza Zali

**Affiliations:** ^1^ Foodborne and Waterborne Diseases Research Center Research Institute for Gastroenterology and Liver Diseases Shahid Beheshti University of Medical Sciences Tehran Iran; ^2^ Gastroenterology and Liver Diseases Research Center Research Institute for Gastroenterology and Liver Diseases Shahid Beheshti University of Medical Sciences Tehran Iran

**Keywords:** antibiotic resistance, Crohn's disease, extraintestinal manifestations, *Klebsiella pneumoniae*, recurrent urinary tract infection, urinary tract stones, urolithiasis

## Abstract

Recurrent urinary tract infections with resistant strains of *Klebsiella pneumoniae* are a potential complication of the long‐term use of immunosuppressive therapy in patients with Crohn's disease.

## Graphical abstract

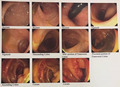

## 1 BACKGROUND

Urolithiasis is one of the extraintestinal manifestations that occurs in 25%–30% of the patients with inflammatory bowel disease (IBD) and can result in persistent urinary tract infection. Development of antibiotic resistance and overgrowth of multidrug‐ resistant (MDR) pathobionts such as *Klebsiella pneumoniae* is favored under the stress of antibiotic pressure.

Crohn's disease (CD) is a type of IBD, and also known as a chronic, progressive, and potentially disabling disease, which generally affects the gastrointestinal tract from mouth to anus.^1^ It is characterized by clinical features of bowel involvement and transmural inflammation. The etiology of CD is currently unknown. However, several factors including aberrant host immune response, genetic predisposition, environmental parameters, and dysbiosis in the gut microbiota contribute to the pathogenesis of CD.^2^, ^3^ The intestinal strictures, fistula formation, and abscesses problems are described as common intestinal complications of CD.^4^ CD patients are usually under treatment with corticosteroids, immunomodulators, and biological agents that weaken host immunity and increase the risk of acquiring opportunistic infections.^3^, ^5^ It is estimated that prevalence of extraintestinal manifestations (EIMs) in IBD differs between 6% and 46%.^6^ The IBD‐associated EIMs can be observed in every organs and involve skin, eyes, joints, biliary tract, liver, and kidneys.^7^ Renal manifestations are often presented in patients with IBD as nephrolithiasis, amyloidosis, tubulointerstitial nephritis, and glomerulonephritis. The pathophysiological mechanisms of renal manifestations have not been clarified yet.^8^ However, it may be due to intestinal inflammatory activity, bacterial overgrowth, or side effects of medical treatment used to control bowel inflammation.^9^, ^10^, ^11^

It is reported that nephrolithiasis is more prevalent in IBD patients (about 25%–30%) compared to general population.^12^ Urinary tract infection (UTI) is the most common complication relating to kidney stone. It is recommended to remove stones and begin antibiotic therapy to eliminate bacterial infections.^13^

Various kinds of antibiotics are prescribed for UTI treatment. Due to overuse and misuse of antibiotics, emergence and development of MDR microorganisms in UTI are becoming a major concern worldwide. The resistance phenomenon leads to treatment failure, and substantially reduces therapeutic options in immunocompromised individuals.^14^, ^15^ Herein, we report a case of CD with recurrent UTI due to MDR *K*. *pneumoniae* for 2 years, which developed resistance against different types of antibiotic over time.

## 2 CASE EXAMINATION

### 2.1 History of present illness

A 53‐year‐old man has presented with a 2‐year history of urinary complications. His medical history was remarkable for CD diagnosed at age 34. He was admitted to the emergency department with intensive abdominal pain in November 2000. In addition, anorexia, nausea, vomiting, diarrhea, and fatigue had been the associated symptoms. The flat and tense abdomen without any masses was observed in his primary physical findings. Maximum tenderness was observed in the right lower quadrant. No hernias were palpated. No significant fistulas or fissures were diagnosed upon rectal examination.

Stool examination for ova, parasites, *Clostridioides* (formerly *Clostridium*) *difficile* toxin, and bacterial pathogens was performed. The results were negative for above tests. Computed tomography (CT) of the abdomen and pelvis revealed bowel thickness at the ileocecal and sigmoid areas which indicated IBD. The appendix was normal without any inflammatory changes. For further diagnostic clarifications, colonoscopy with biopsy analysis was recommended. The abdominal transient radiography and colonoscopy findings revealed multiple long deep ulcers in the ileum and ileocecal valves with normal mucosa without lymphadenopathy in the entire of colon. CD in the terminal ileum was diagnosed by histologic findings.

### 2.2 Past treatment history and follow‐up

**FIGURE 1:**
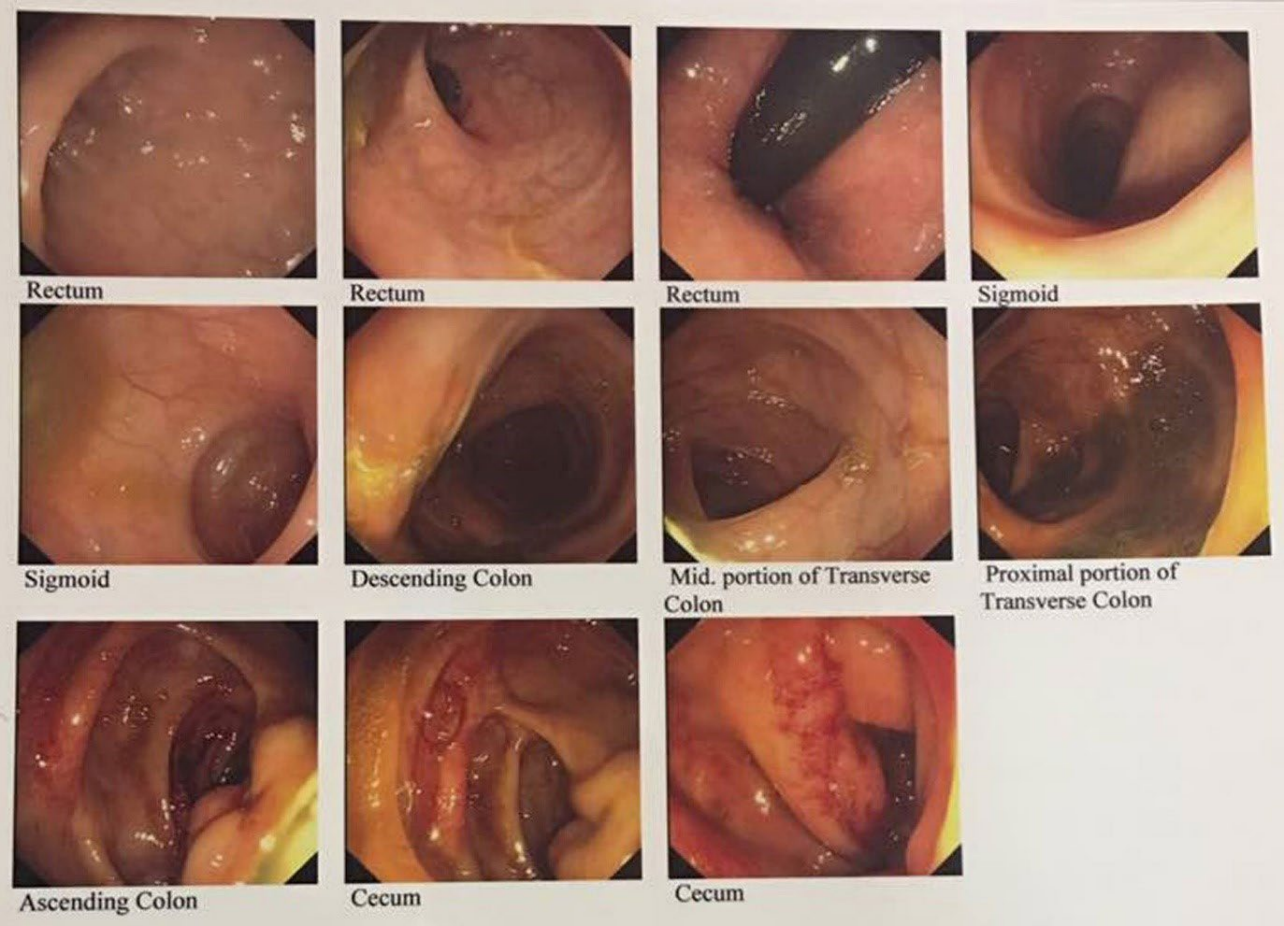
Images obtained through colonoscopy of the patient diagnosed with Crohn's disease (CD). As seen, there was diffuse erythema with ulceration and bruisability in cecum and terminal ileum

Mesalazine and prednisolone were prescribed for the relief of his recurrent abdominal pain. Overall, his symptoms and nutritional status improved, resulting in clinical remission for 2 years. In September 2002, the patient presented to emergency department with fever and abdominal pain. Besides fever, the inflammatory markers were increased with white blood cells (WBC) count of 17.9 × 10^9^/L, C‐reactive protein (CRP) of 190 mg/L and erythrocyte sedimentation rate (ESR) of 44 mm/h. The ultrasound imaging revealed abscess at the distal ileum, which was treated with metronidazole and ciprofloxacin antibiotics. After 6 months, he was administrated to hospital with acute inflammation and flare‐up symptoms. The patient was diagnosed with recurrent abscess at the distal ileum by abdominal CT scan. At this time, the percutaneous aspiration was done, and metronidazole, gemifloxacin, and intravenous ceftriaxone were taken to reduce harmful intestinal bacteria. The immunosuppressive therapy started with azathioprine. He was under supervision by a gastroenterologist for follow‐up and reassessment of medical therapy. In 2006, the symptoms worsened again, and the patient presented to hospital with gastric pain for further follow‐up. Colonoscopy demonstrated mild erythematous mucosa with segmental extension in the sigmoid colon. The internal hemorrhoids without bleeding were observed. Stricture of the terminal ileum was also detected. Treatment with mesalazine and ketorolac relieved gastric complication. In June 2009, the patient referred to hospital with recurring right lower quadrant pain, abdominal bloating, and increased intestinal gas. He was admitted for colonoscopy procedure. The results showed terminal ileum stricture, and mild scattered colitis of cecum was observed as well (Figure 1). Abdominal CT scan revealed fistulating distal ileum with abscess formation. Treatment with infliximab with 6‐mercaptopurine was initiated after surgical incision and drainage of the abscess. The patient was in remission stage for 5 years.

In May 2014, the magnetic resonance imaging (MRI) of abdomen represented that a round mass with 15 mm diameter was evident between cecum and anterior abdominal wall suggestive of granulation tissue formation. Kidney stones were also detected. The patient with colic cramps, fever, and touchable mass at right lower quadrant was admitted to hospital for surgery and excision of the fistulous tract. The ileectomy was done by laparoscopic procedure. Histology of the resected specimen showed a purulent inflammatory mass. Medical immunosuppressive therapy was as previous with addition of levofloxacin, metronidazole, and cefotaxime antibiotics. After his convalesce, he was referred for extracorporeal shock wave therapy to treat kidney stones and was treated with ciprofloxacin and ceftazidime to control bacterial growth.

After 3 years, the abdominal CT showed a mass lesion involved in the long segment of cecum, ascending colon, and terminal colon. The patient was referred to physician in order to survey further clinical aspects. At this time, infliximab was stopped and prednisolone plus adalimumab were prescribed. Soon after an uneventful time of 3 months, he re‐admitted to emergency department with acute UTI.

### 2.3 Laboratory examination and antimicrobial susceptibility testing

**FIGURE 2:**
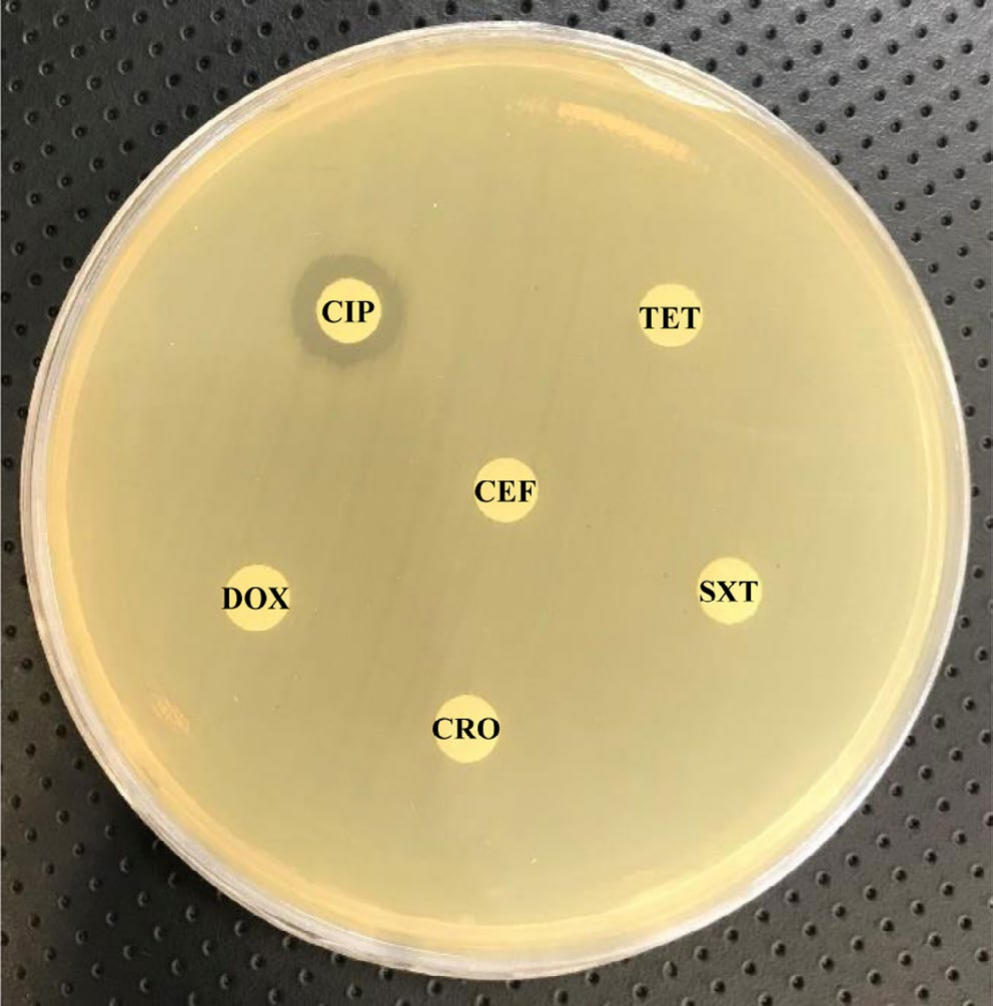
A representative antibiogram image of *K*. *pneumonia* isolate showing resistance to ciprofloxacin (CIP), tetracycline (TET), cephalothin (CEF), doxycycline (DOX), cotrimoxazole (SXT), and ceftriaxone (CRO) antibiotics investigated

Urine analysis revealed 4–6 WBCs per high‐power field, and 3–4 RBCs per high‐power field with no granular and hyaline casts. The microbiologic tests detected *K*. *pneumoniae* (>10^6^ CFU/ml of urine) in urine culture. Antimicrobial susceptibility of *K*. *pneumoniae* was done using disk diffusion method according to CLSI guideline.^16^ The antimicrobial agents tested were as follows: tetracycline (30 µg), nitrofurantoin (300 µg), chloramphenicol (30 µg), gentamicin (10 µg), ciprofloxacin (5 µg), levofloxacin (5 µg), ofloxacin (5 µg), cephalothin (30 µg), doxycycline (30 µg), cotrimoxazole (25 µg), ceftriaxone (30 µg), cefazolin (30 µg), cefoxitin (30 µg), amikacin (30 µg), imipenem (10 µg), piperacillin (100 µg), and cefepime (30 µg) (Rosco). The bacterial isolate was resistant to ciprofloxacin, tetracycline, cephalothin, doxycycline, cotrimoxazole, and ceftriaxone (Figure 2). According to the patient's history, ceftazidime was approved for treatment. The urinary infection symptoms were decreased for 3 months. After that recurrent infection was occurred with *K*. *pneumoniae*, which was sensitive to nitrofurantoin, chloramphenicol, levofloxacin, and ceftizoxime. Treatment with levofloxacin (500 mg) was continued for 2 weeks, and the symptoms were decreased for 2 months. After this period, UTI recurred and levofloxacin was continued based on antibiotic susceptibility profile of the isolate. Regardless, after 4 months treatment failure occurred. Thus, antibiotic therapy was shifted to cefepime according to the susceptibility testing. He was followed up every month for urine culture and urine analysis. The bacterial cultures were negative for 5 months. After all, recurrent UTI symptoms were confirmed by standard laboratory tests. The *K*. *pneumoniae* with ≥10^2^ CFU/ml of urine was reported. The ultrasound imaging exhibited no prostate, urinary bladder, ureter, or kidneys abnormalities. Particularly, no abnormal cysts and enterovesical fistulas were found in the urinary bladder and kidneys. The nephrolithiasis was not reported. Following unsuccessful treatment and persistent infection, he was treated with intravenous meropenem (500 mg) for 20 days. The UTI symptoms were improved after 3 months. Additional antimicrobial tests including ofloxacin (5 µg), ceftizoxime (30 µg), and gemifloxacin (5 µg) were asked. The results showed that bacterial isolate was resistant to all types of antibiotics except nitrofurantoin, ceftizoxime, and gemifloxacin. The infection was controlled for 2 months by gemifloxacin. In addition, according to the recurrent infection, the adalimumab was discontinued and nitrofurantoin (100 mg) was prescribed for a month, which resulted in a rapid improvement of the patient's conditions. To reduce inflammatory symptoms, the medical treatment shifted to dexamethasone (0.5 mg), azathioprine (50 mg), and mesalazine (500 mg). The patient was re‐evaluated after 1 month without any UTI symptoms and remains well until now. The patient's timeline for the main clinical symptoms, IBD medications, and antibiotic resistance profile of the isolated *K*. *pneumoniae* is presented in Table S1.

## 3 DISCUSSION

Urinary tract complications are well defined as an EIM of CD. The pathologic effects of CD on urinary tract are presented as inflammatory (fistula formation, perivesical abscess, cystitis, and ureteral obstruction) or metabolic disorders.^17^, ^18^ It is estimated that prevalence of urinary tract stones in CD patients is higher than general population.^19^ Stone formation is associated with chronic dehydration and loss of alkaline intestinal fluid that results in a highly concentrated and acidic urine, and consequently leads to formation of uric acid stone in CD patients.^18^ Accordingly, urolithiasis is one of the most common EIM in these patients that can result in recurrent UTI.^11^, ^20^ Moreover, the prevalence of urolithiasis in patients with small bowel surgery is often more than other individuals.^21^

It has been reported that IBD patients treated with infliximab, prednisolone, and immunomodulatory drugs are at higher risk for opportunistic and serious infections.^22^, ^23^ *K*. *pneumoniae* has been emerged as a prominent opportunistic pathogen that causes a wide range of infections, including pneumonia, UTI, bloodstream infections, and pyogenic liver abscess.^24^ Classically, these infections are associated with hospitalization or otherwise immunocompromised individuals, and are routinely treated with β‐lactam antibiotics and other antimicrobials effective against enteric bacterial agents.^25^ Furthermore, hypervirulent strains of *K*. *pneumoniae* have become increasingly resistant to a wide range of antibiotics and have acquired additional genetic traits, rendering infection by these microorganisms very difficult to treat.^26^ The recommended therapeutic strategy is the complete stone clearance followed by specific antibiotic treatment. However, persistent infections can occur due to existence of residual stones that facilitate rapid formation of the stones which may lead to failed antibiotic therapy.^27^ MDR *K*. *pneumoniae* strains have significantly increased in recent years. *K*. *pneumoniae* exploits different strategies to evade the antimicrobial activity of antibiotics. The production of extended‐spectrum β‐lactamases (ESBLs) and cephalosporinases is considered as one of the main mechanisms involved in the drug resistance of *K*. *pneumoniae* strains.^28^ Also, efflux pumps play a major role in emerging MDR phenotypes in several Gram‐negative bacteria particularly *K*. *pneumoniae*, allowing the extrusion of toxic substrates including virtually all classes of clinically relevant antibiotics out of cells. Moreover, other resistance mechanisms, including natural variations or acquired changes in the target sites of antimicrobials, and the capability of bacteria to develop biofilm structures may be engaged in resistance to antimicrobial compounds.^29^

The massive use and inappropriate choice of antibiotics are considered as the most important factors for development of bacterial resistance to antimicrobial agents.^14^, ^30^ Several reports have shown that antimicrobial resistance is a major challenge in treatment of UTIs and related disorders.^31^, ^32^, ^33^, ^34^, ^35^ In this case, the patient with CD history was followed for recurrent UTI for 2 years. The isolated *K*. *pneumoniae* strain showed resistance to different types of antibiotics, which is supposed to be developed because of overconsumption of antibiotic agents during the long period of complication. It has been confirmed that prolonged antibiotic therapy can lead to development of antibiotic resistance in a microorganism which originally was sensitive, and also can alter the composition of host intestinal microbiota.^36^ Furthermore, antibiotic resistance can occur via genetic transfer of resistant genes, inactivation or modification of antibiotics, modification of antibiotic targets, and reduced outer membrane permeability of antibiotics through efflux‐mediated extrusion of drugs.^37^, ^38^

Corticosteroids and immunomodulators have extensive impacts on the immune system, and patients treated with such drugs may be at an increased risk for serious infections. In addition, use of anti‐TNFα medications such as infliximab and adalimumab have been associated with an elevated risk of serious infections in patients with IBD.^39^, ^40^, ^41^ In conclusion, we suggest that emergence of recurrent UTI by *K*. *pneumoniae* possibly associates with the long‐term use of IBD medications in the CD patient presented in this report. Taken together, since CD is a chronic inflammatory disease requiring ongoing treatments, the increased risk for getting infections during IBD therapies should be deeply considered.

## CONFLICT OF INTEREST

None declared.

## AUTHOR CONTRIBUTION

MA performed the microbiological examinations and antimicrobial susceptibility testing. AY and MT reviewed the literature and wrote the manuscript. MRZ performed the colonoscopy and critically revised the manuscript. We also confirm that all authors have read and approved the final version of the manuscript.

## ETHICAL APPROVAL AND CONSENT TO PARTICIPATE

The study protocol was approved by the Ethical Review Committee of RIGLD at Shahid Beheshti University of Medical Sciences (Project No. IR.SBMU.RIGLD.REC.1396.185). All experiments were performed in accordance with relevant guidelines and regulations recommended by the institution.

## CONSENT FOR PUBLICATION

Written informed consent was obtained from the patient for publication of this case report and any accompanying images. A copy of the written consent is available for review by the Editor in Chief of this journal upon request.

## Supporting information

Table S1

## Data Availability

All data generated or analyzed during this study are included in this published paper.
