# Supplementary material for: Recurrent urinary tract infection with antibiotic‐resistant Klebsiella pneumoniae in a patient with Crohn's disease: A case report
Source: Clin Case Rep. 2021 Aug 11;9(8):e04531. doi: 10.1002/ccr3.4531 (PMC8355749; doi:10.1002/ccr3.4531)
Supplement: Supplementary file 1 — Table S1 [file CCR3-9-e04531-s001.docx]

**Supplementary Table 1.** The patient’s timeline for the main clinical symptoms, IBD medications and antibiotic resistance profile of the isolated *K. pneumoniae*.

| **Date** | **Main clinical symptoms** | **Antibiotic resistance profile of *K. pneumoniae* isolate** | **IBD medications used** |
| --- | --- | --- | --- |
| 2000 | Intensive abdominal pain | TET, CIP, CEF, DOX, SXT, CRO | Mesalazine and prednisolone |
| 2002 | Fever and abdominal pain and IBD flare-up | TET, CIP, CEF, DOX, SXT, CRO, GEN, CAZ | Azathioprine |
| 2006 | IBD symptoms worsened, gastric pain | TET, CIP, CEF, DOX, SXT, CRO, GEN, CAZ | Mesalazine and ketorolac |
| 2009 | Recurring right lower quadrant pain, abdominal bloating and increased intestinal gas | TET, CIP, CEF, DOX, SXT, CRO, GEN, CAZ | Infliximab and 6-mercaptopurine |
| 2014 | Colic cramps, fever and kidney stones | TET, CIP, CEF, DOX, SXT, CRO, GEN, CAZ | Infliximab and 6-mercaptopurine |
| 2017 | Fever and abdominal pain and IBD flare-up | TET, CIP, CEF, DOX, SXT, CRO, GEN, CAZ, CHL, LVX, OFX | Prednisolone |

IBD, inflammatory bowel disease; *K. pneumoniae*; *Klebsiella pneumoniae*, TET, tetracycline; CIP, ciprofloxacin; CEF, cephalothin; DOX, doxycycline; SXT, cotrimoxazole; CRO, ceftriaxone; GEN, gentamicin; CAZ, ceftazidime; CHL, Chloramphenicol; LVX, levofloxacin; OFX, Ofloxacin.
